# Severe injuries in competitive cycling during an olympic cycle: injury patterns, time loss, and return-to-performance outcomes

**DOI:** 10.1186/s13102-026-02201-x

**Published:** 2026-11-03

**Authors:** Christophe Lambert, Maxime Lambert, Marlene Galandi, Bernd Wolfarth, Casper Grim, Sven Shafizadeh, Ramona Ritzmann

**Affiliations:** 1 Department of Trauma and Orthopaedic Surgery, University of Witten/Herdecke, Cologne Merheim Medical Centre, Cologne, Germany; 2 Department of Biomechanics, University of Freiburg, Freiburg, Germany; 3 Department of Sports Medicine, Charité – University Medical Center Berlin, corporate member of Freie Universität Berlin and Humboldt-Universität zu Berlin, Berlin, Germany; 4 Department of Orthopedic Surgery and Sports Traumatology, Sana Medical Centre, Witten/Herdecke University, Cologne, Germany; 5 Department of Orthopedic, Trauma and Hand Surgery, Klinikum Osnabrück, Osnabrück, Germany; 6 Orthopädie em Veedel, Praxis für Orthopädie, Unfallchirurgie und Sportmedizin, Cologne, Germany; 7 Charité – Universitätsmedizin Berlin, corporate member of Freie Universität Berlin and Humboldt-Universität zu Berlin, Berlin, Germany

**Keywords:** Epidemiology, Injury patterns, Road cycling, Mountain biking, Track cycling, BMX, Time loss, Upper extremity injuries, Fractures, Return to sport, Rehabilitation

## Abstract

**Background:**

Cycling is an established Olympic sport associated with a distinct injury profile that has not yet been comprehensively investigated. This study aimed to retrospectively assess and analyze severe cycling-related injuries resulting in ≥ 3 weeks of time loss over a complete Olympic cycle.

**Methods:**

A total of 192 cyclists were included, based on a retrospective online questionnaire assessing injury location, type, context, time loss, and return-to-sport outcomes. Comparisons by sex, discipline, and competitive level were performed at the athlete level using exact tests with 95% confidence intervals and were considered exploratory.

**Results:**

Seventy-two of 192 athletes sustained at least one severe injury over the five-year period, corresponding to a proportion of 37.5% (95% CI 31.0–44.5). A total of 237 injuries were recorded, with the upper extremity (33.8%) and lower extremity (27.8%) being the most frequently affected regions. The most common diagnoses were hand fractures (*n* = 14), clavicle fractures (*n* = 13), and non-specific knee injuries (*n* = 9). Hand fractures (33.3%; *p* = 0.002) and shoulder dislocations (20.0%; *p* = 0.002) were most frequent among BMX athletes, while hand fractures also occurred more frequently in competitive athletes than in recreational athletes (13.5% vs. 1.0%; *p* = 0.001). Clavicle fractures occurred predominantly during competition and non-specific knee injuries during training. Mean time loss ranged from 6.5 weeks for hand fractures to 13.3 weeks for shoulder dislocations. Full return to pre-injury performance ranged from 85.7% after hand fractures to 28.6% after acromioclavicular (AC) joint separations.

**Conclusion:**

Injury patterns differed by discipline and setting, suggesting that prevention strategies should address both. The low rate of full return to performance after AC joint separations despite moderate time loss underlines the importance of distinguishing return to sport from return to performance in rehabilitation.

**Supplementary Information:**

The online version contains supplementary material available at https://doi.org/10.1186/s13102-026-02201-x.

## Introduction

Cycling is an established Olympic sport with worldwide participation and increasing societal relevance. The Olympic cycling program comprises multiple disciplines, including road cycling, track cycling, mountain bike cross-country, BMX racing, and BMX freestyle, which differ in competition format, environmental conditions, technical demands, and biomechanical loading patterns [1].

Despite growing research interest, the epidemiology of cycling-related injuries remains incompletely characterized. Available studies indicate substantial discipline-specific differences in injury risk, with BMX and mountain biking demonstrating higher injury rates compared with road and track cycling during Olympic competitions [2, 3]. Reported injury patterns include acute traumatic injuries, particularly fractures and shoulder injuries, as well as overuse conditions associated with repetitive mechanical loading and prolonged cycling positions [4, 5]. However, comparisons between studies remain challenging due to heterogeneous methodologies, inconsistent injury definitions, and varying classification systems [4]. To improve comparability, standardized methods for recording and reporting sports injury data have been proposed, including consensus definitions of injury severity [6]. In the present study, severe injuries were defined a priori as those causing at least three weeks of time loss, as described in the Methods. Because the mode of onset was not recorded, injuries are reported according to whether they occurred in training or competition and are not classified as traumatic or overuse.

Prospective data from professional road cycling demonstrate that acute traumatic injuries represent a major cause of time loss, with fractures contributing substantially to injury burden [7]. In mountain biking, large-scale surveillance studies have identified the head, wrist/hand, and shoulder as frequently affected regions, with differences observed between male and female athletes [8]. Similarly, recent reviews of BMX highlight the importance of discipline-specific injury mechanisms related to falls, jumps, and collisions [9]. Nevertheless, available literature rarely provides a comprehensive analysis of severe injuries across cycling disciplines while simultaneously considering sex, performance level, injury context, time loss, and return-to-performance outcomes.

A detailed understanding of severe injuries and their consequences is essential for developing targeted prevention strategies and optimizing medical management in cycling. This study therefore examined severe cycling-related injuries over one complete Olympic cycle from 2012 to 2016. The analysis covered the Olympic cycling disciplines, namely road cycling, track cycling, mountain biking, and BMX, and included cyclists across the full performance spectrum, from recreational to international level. This broad scope was intentional, as it provides an overview of severe injuries in cycling and allows a comparison between competitive and recreational athletes. Specifically, we investigated the proportion of injured athletes, injury patterns, differences by sex, discipline, competitive level, injury setting, time loss, and return-to-performance outcomes.

## Methods

### Study design and setting

This retrospective cross-sectional study investigated the occurrence and characteristics of sports injuries sustained during one Olympic cycle, from the 2012 Summer Olympic Games in London to the 2016 Summer Olympic Games in Rio de Janeiro. Self-reported injuries were analyzed with regard to their anatomical location, injured tissue, time loss, and subsequent return to sport.

### Survey instrument and recruitment

Data were collected through an online questionnaire (SurveyMonkey) between July 2016 and August 2017 as part of a multi-sport survey covering all Olympic Sports [10]. Participants reported injuries sustained during the 2012 to 2016 Olympic cycle separately for each calendar year. The 139-item instrument was based on a previously developed and validated questionnaire [11], adapted in collaboration with the German Olympic Sports Confederation, and comprised three sections (Supplementary Appendix). The first section recorded the main cycling discipline, the competitive level, and average weekly training hours. The second recorded injuries, including the affected anatomical region, diagnosis, injured structure, setting (training or competition), time loss in weeks, and performance level after return to sport. The third section recorded sociodemographic characteristics. The survey was disseminated through the project website, social media, and national cycling federations, which forwarded it to their members. Because recruitment was open, the number of athletes reached could not be determined and a response rate could not be calculated. 

### Study population and eligibility

**Fig. 1:**
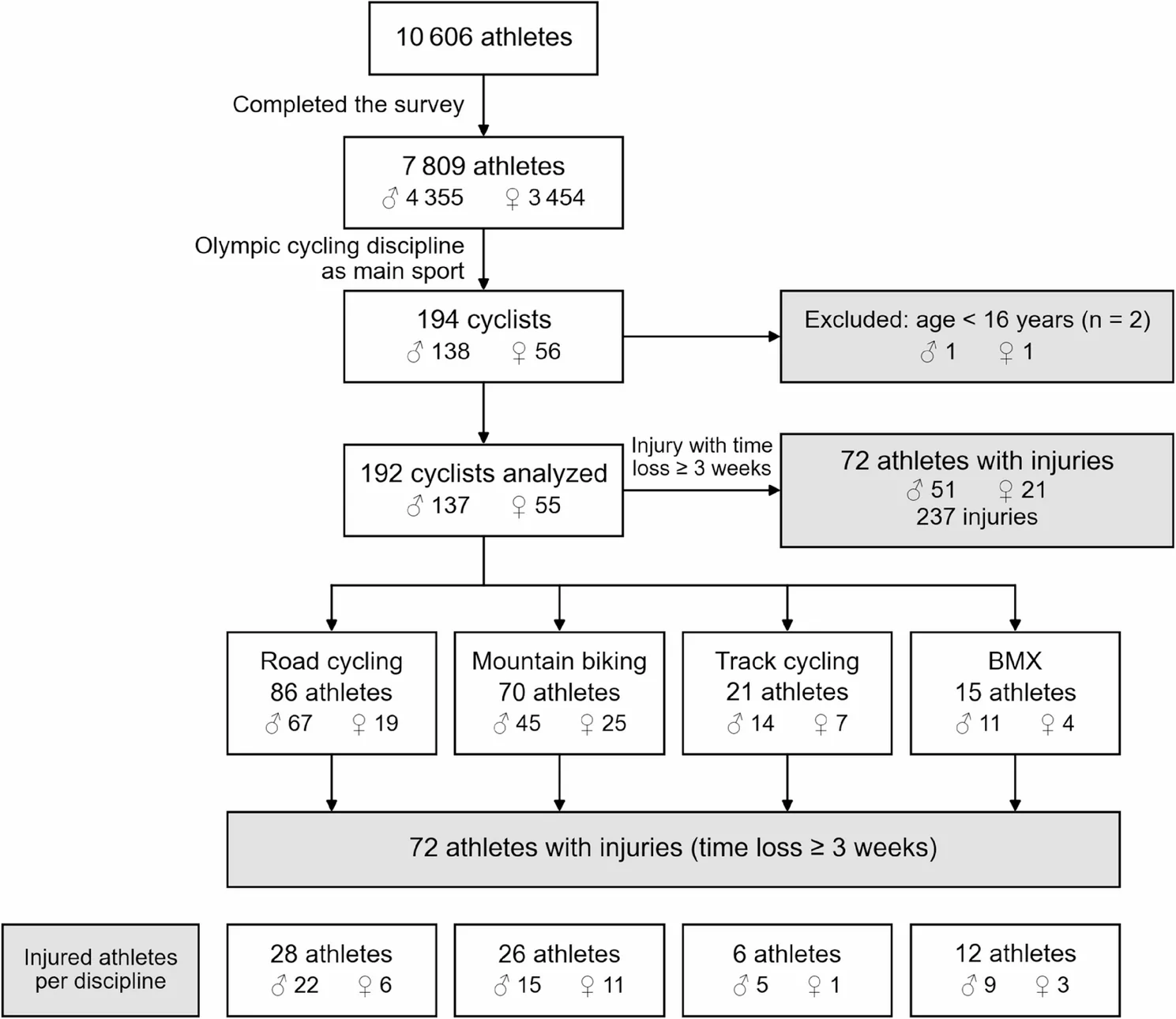
Participant flow. The questionnaire was part of a multi-sport survey; the numbers of athletes who started and completed the survey refer to all sports. Because recruitment was open, the number of athletes reached and a response rate could not be determined. ♂ male, ♀ female

Of 10,606 athletes who started the questionnaire, 7,809 (73.6%) completed it, and 194 of these reported an Olympic cycling discipline as their main sport (Fig. 1). Eligible participants were cyclists who were active between 2012 and 2016 and who provided complete data on the primary variables, namely time loss and cycling discipline. The complete-case approach applied to these primary variables, whereas individual secondary items, such as average weekly training hours, could be missing without leading to exclusion. Participants under 16 years were excluded from the analysis because written consent from a parent or legal guardian, which is required for participants of this age, was not available. In total, 192 cyclists were included in the analysis (Fig. 1). For discipline-specific analyses, participants were categorized into road cycling, mountain biking, track cycling, and BMX. For analyses by competitive level, athletes who reported membership in a national or regional squad (A, B, C, or D/C squad) or competition in the national league (Bundesliga) in at least one year were classified as competitive athletes. All other participants, including recreational athletes, sports students, coaches, and former athletes, were classified as recreational.

### Definition of injuries and return to sport

A qualifying (severe) injury was defined a priori as any self-reported injury sustained during cycling training or competition that led to a time loss of at least three weeks. Time loss was recorded as the self-reported number of weeks during which the athlete was unable to compete in their sport because of the injury. This corresponds to the most severe category of the time-loss classification proposed by Olsen et al. [12]. Time losses reported as exactly three weeks were retained, as they most likely reflect rounding. The mode of onset, whether acute or gradual, was not assessed, so injuries are reported according to whether they occurred in training or competition rather than classified as traumatic or overuse. After returning to sport, athletes reported their performance level relative to the pre-injury level as full, slightly reduced, or markedly reduced; the fourth response option, discontinuation of the sport, was not selected for any of the analyzed diagnoses. This referred to the level reached after completed rehabilitation, and no fixed post-injury time point was applied. 

### Ethical approval

The study followed the ethical principles of the Declaration of Helsinki and was approved by the Ethics Committee of Witten/Herdecke University (No. 154/2017). Before starting the questionnaire, all participants were informed about the aims of the study and the anonymous, aggregated use of their data and gave their informed consent electronically.

### Statistical analysis

Statistical analyses were performed using IBM SPSS Statistics (version 29; IBM Corp., Armonk, NY, USA). Python (version 3.13) was used to reproduce the SPSS results independently and to generate the figures. Continuous variables are presented as means with standard deviations (SD), medians, and ranges, and categorical variables as frequencies and percentages. The athlete was the unit of analysis for all group comparisons, which were performed separately for each diagnosis, whereas injury counts are reported to describe the injury spectrum. Each athlete was therefore counted once per diagnosis, and because a few athletes sustained the same injury in more than one year, these athlete-based counts can be slightly lower than the injury counts reported in the Results. Subgroup analyses focused on the most frequently reported diagnoses. Because four diagnoses were tied at seven cases, a cut-off of at least seven cases was applied to avoid arbitrarily splitting this tie, which yielded seven diagnoses. These were analyzed with respect to sex, cycling discipline, competitive level, injury context, time loss, and return to performance. Subgroup results are reported as the proportion of athletes within each subgroup, together with 95% confidence intervals calculated using the Wilson method. Because time-loss data were skewed, 95% confidence intervals for mean time loss were estimated using bias-corrected and accelerated bootstrapping. Pearson's chi-square test was used to compare the overall proportion of injured athletes between sexes. Fisher's exact test was applied for 2 × 2 contingency tables, and the Fisher–Freeman–Halton exact test for larger contingency tables. Given the small number of events and the multiple comparisons, all subgroup analyses are considered exploratory. Statistical significance was defined as a two-sided p-value <0.05.

## Results

A total of 192 cyclists were included in the study. Participant characteristics are summarized in Table 1. During the five-year study period, 72 of 192 athletes reported at least one severe injury, defined by at least three weeks of time loss, which corresponds to 37.5% of respondents (95% CI 31.0–44.5) and a total of 237 reported injuries. Injury prevalence did not differ significantly between male and female athletes (37.2% vs. 38.2%, *p* = 0.902). Road cycling represented the largest discipline (44.8%), followed by mountain biking (36.5%), track cycling (10.9%), and BMX (7.8%). Half of the athletes were competitive (*n* = 96) and half recreational (*n* = 96).

**Table 1:** Participant characteristics and injury status

Variable	Category	Total cohort (*n* = 192)
Sex	Male	137 (71.4%)
	Female	55 (28.6%)
Age (years)	Mean ± SD	32.3 ± 13.9
	Range	16–70
Level of competition	Competitive	96 (50%)
	Recreational	96 (50%)
Primary cycling discipline	Road cycling	86 (44.8%)
	Mountain biking	70 (36.5%)
	Track cycling	21 (10.9%)
	BMX	15 (7.8%)
Injury status	Injured	72 (37.5%)
	Not injured	120 (62.5%)
Total injuries reported		237

*SD *standard deviation, Injured at least one severe injury (time loss ≥ 3 weeks) between 2012 and 2016. Total injuries reported include multiple injuries per athlete

### Injured body region

The upper extremity was the most frequently affected body region, accounting for 33.8% of all reported injuries (80/237). The arm was the most frequently injured anatomical region (n = 42; 17.7%), followed by the leg and foot excluding the knee (n = 38; 16.0%), and the shoulder (n = 38; 16.0%). Overall, injuries to the lower extremity including the knee accounted for 27.8% of all reported injuries (66/237).

### Most frequent injuries

The most frequently reported injury was hand fracture (n = 14), followed by clavicle fracture (n = 13) and non-specific knee injury (n = 9). Mild traumatic brain injury (concussion), shoulder dislocation, acromioclavicular (AC) joint separation, and forearm fracture each ranked fourth, with seven cases reported.

### Most common injury diagnoses by sex

**Fig. 2:**
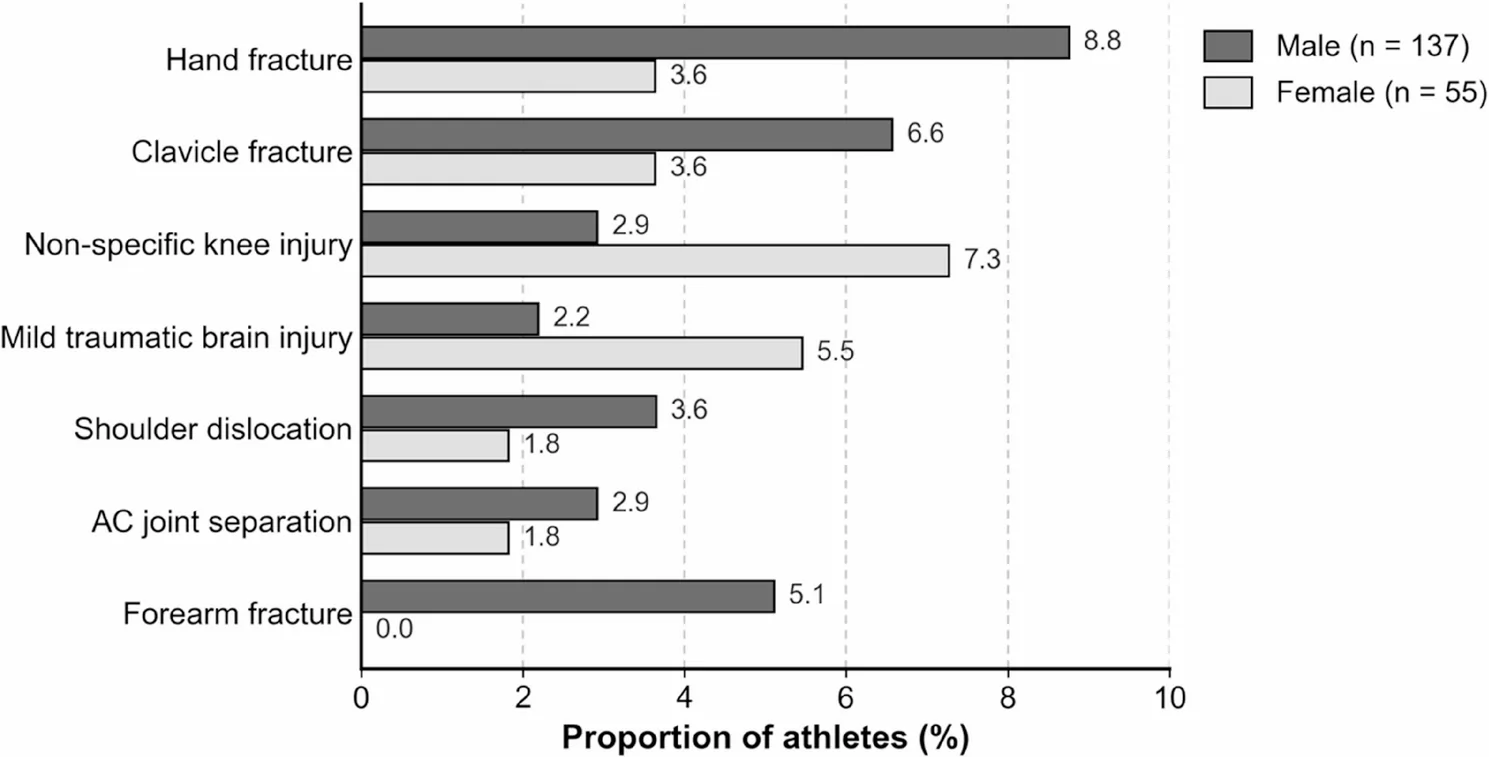
Proportion of athletes with each of the seven most common diagnoses by sex. Values are percentages of all female (n = 55) and male (n = 137) athletes; each athlete was counted once per diagnosis. No significant sex differences were observed (Fisher’s exact test, all p > 0.05).

No significant sex differences were observed for any of the seven most common diagnoses when injuries were expressed as the proportion of athletes within each sex (Fig. 2). Hand fractures occurred in 12 of 137 male athletes (8.8%, 95% CI 5.1–14.7) and 2 of 55 female athletes (3.6%, 95% CI 1.0–12.3; p = 0.357), and clavicle fractures in 9 of 137 males (6.6%, 95% CI 3.5–12.0) and 2 of 55 females (3.6%; p = 0.732). Non-specific knee injuries were more frequent in female athletes (4 of 55, 7.3%) than in male athletes (4 of 137, 2.9%; p = 0.229). Forearm fractures occurred only in male athletes (7 of 137, 5.1%; p = 0.195). No significant sex differences were found for mild traumatic brain injuries (p = 0.356), shoulder dislocations (p = 0.676), or AC joint separations (p = 1.000).

### Most frequent injuries by cycling discipline

**Fig. 3:**
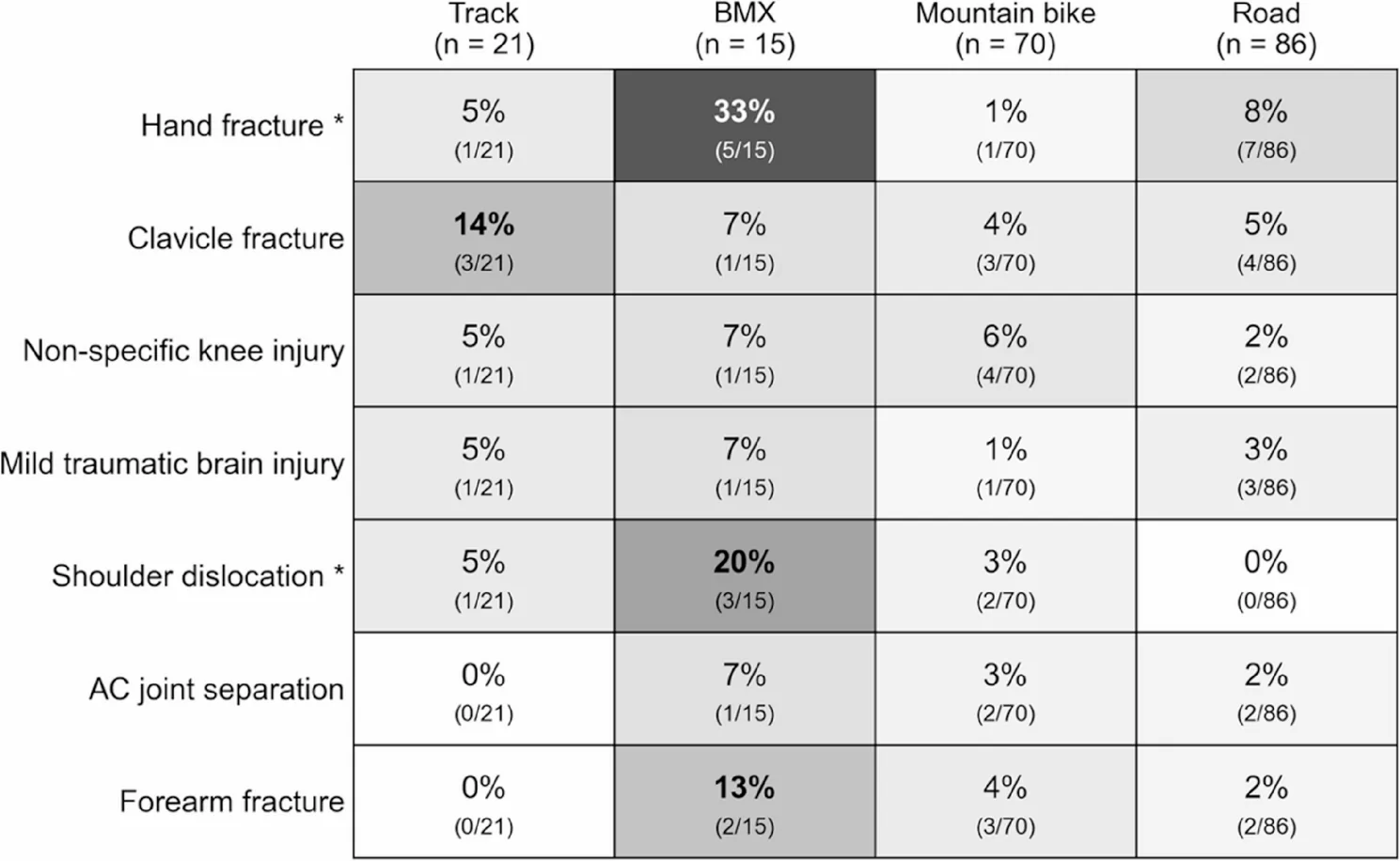
Proportion of athletes with each of the seven most common diagnoses within each cycling discipline. Each cell shows the percentage of all athletes in the respective discipline with the diagnosis and, in parentheses, the number of affected athletes and the discipline size; darker shading indicates a higher proportion. Each athlete was counted once per diagnosis. *p < 0.05 (Fisher-Freeman-Halton exact test; hand fracture and shoulder dislocation, both p = 0.002)

Because the disciplines differed considerably in size, diagnoses are reported as the proportion of athletes within each discipline (Fig. 3). Hand fractures and shoulder dislocations differed significantly between disciplines. Hand fractures were most frequent in BMX athletes (5 of 15, 33.3%, 95% CI 15.2–58.3), followed by road (7 of 86, 8.1%, 95% CI 4.0–15.9), track (1 of 21, 4.8%), and mountain biking (1 of 70, 1.4%) (p = 0.002). Shoulder dislocations were most frequent in BMX (3 of 15, 20.0%, 95% CI 7.0–45.2), followed by track cycling (1 of 21, 4.8%) and mountain biking (2 of 70, 2.9%), and did not occur in road cycling (0 of 86) (p = 0.002). Clavicle fractures did not differ significantly between disciplines and occurred in 3 of 21 track (14.3%), 1 of 15 BMX athletes (6.7%), 4 of 86 road (4.7%), and 3 of 70 mountain biking (4.3%) (p = 0.245). No significant differences were found for the remaining diagnoses (non-specific knee injuries, p = 0.432; mild traumatic brain injuries, p = 0.401; AC joint separations, p = 0.548; and forearm fractures, p = 0.179; Fig. 3).

### Competition versus training setting

**Fig. 4:**
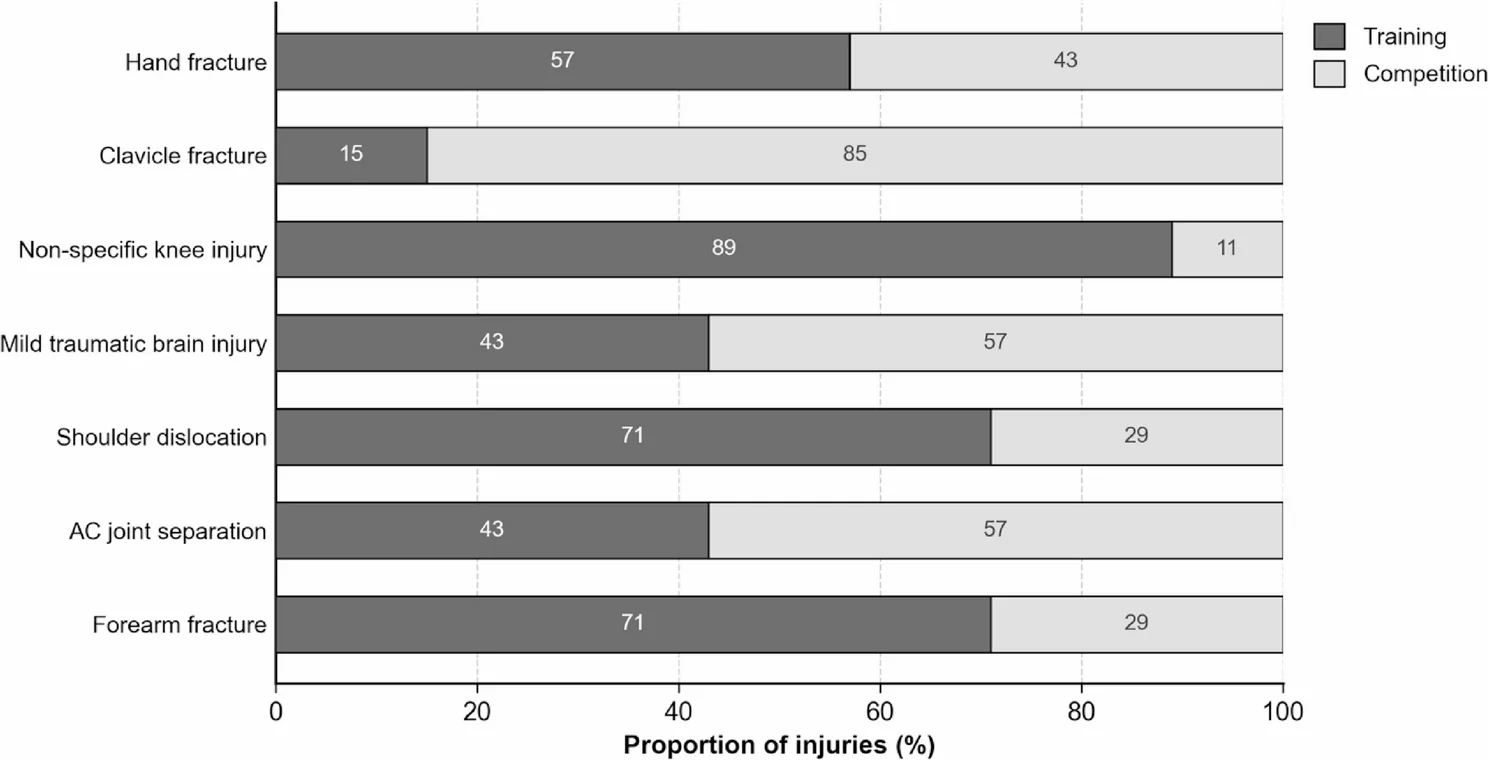
Setting in which the most common diagnoses occurred (training vs. competition). Values are percentages of all injuries with the respective diagnosis (hand fracture n = 14, clavicle fracture n = 13, non-specific knee injury n = 9, mild traumatic brain injury, shoulder dislocation, AC joint separation, and forearm fracture n = 7 each)

Clavicle fractures occurred predominantly during competition, with 84.6% (n = 11) sustained during races (Fig. 4). Mild traumatic brain injuries and AC joint separations also showed a tendency to occur more frequently in competition, with 57.1% (n = 4) of each injury type reported during races. In contrast, several injury types were primarily associated with training. This was most evident for non-specific knee injuries, of which 88.9% (n = 8) occurred during training sessions. Shoulder dislocations (71.4%; n = 5), forearm fractures (71.4%; n = 5), and hand fractures (57.1%; n = 8) were likewise sustained predominantly during training.

### Injury distribution among competitive and recreational athletes

When expressed as the proportion of athletes within each competitive stratum, hand fractures were significantly more frequent in competitive athletes (13 of 96, 13.5%, 95% CI 8.1–21.8) than in recreational athletes (1 of 96, 1.0%, 95% CI 0.2–5.7; p = 0.001; Table 2). None of the other diagnoses differed significantly between competitive levels. Forearm fractures (6 of 96 vs 1 of 96; p = 0.118), clavicle fractures (8 of 96 vs 3 of 96; p = 0.213), mild traumatic brain injuries (4 of 96 vs 2 of 96; p = 0.683), and AC joint separations (3 of 96 vs 2 of 96; p = 1.000) were numerically more frequent in competitive athletes, whereas non-specific knee injuries were more frequent in recreational athletes (5 of 96 vs 3 of 96; p = 0.721). Shoulder dislocations were equally distributed between the groups (3 of 96 in each; p = 1.000).

**Table 2:** Distribution of injuries according to competitive level

Injury type	Competitive athletes (*n* = 96), n (%)	Recreational athletes (*n* = 96), n (%)	*p*-value
Hand fracture	13 (13.5)	1 (1.0)	0.001*
Clavicle fracture	8 (8.3)	3 (3.1)	0.213
Non-specific knee injury	3 (3.1)	5 (5.2)	0.721
Mild traumatic brain injury	4 (4.2)	2 (2.1)	0.683
Shoulder dislocation	3 (3.1)	3 (3.1)	1.000
Acromioclavicular joint separation	3 (3.1)	2 (2.1)	1.000
Forearm fracture	6 (6.3)	1 (1.0)	0.118

Data are presented as the number of athletes with the respective diagnosis and the percentage of all athletes in the group (*n* = 96 each). Each athlete was counted once per diagnosis. P-values were calculated using Fisher’s exact test

*p < 0.05

### Injury-related time loss

**Fig. 5:**
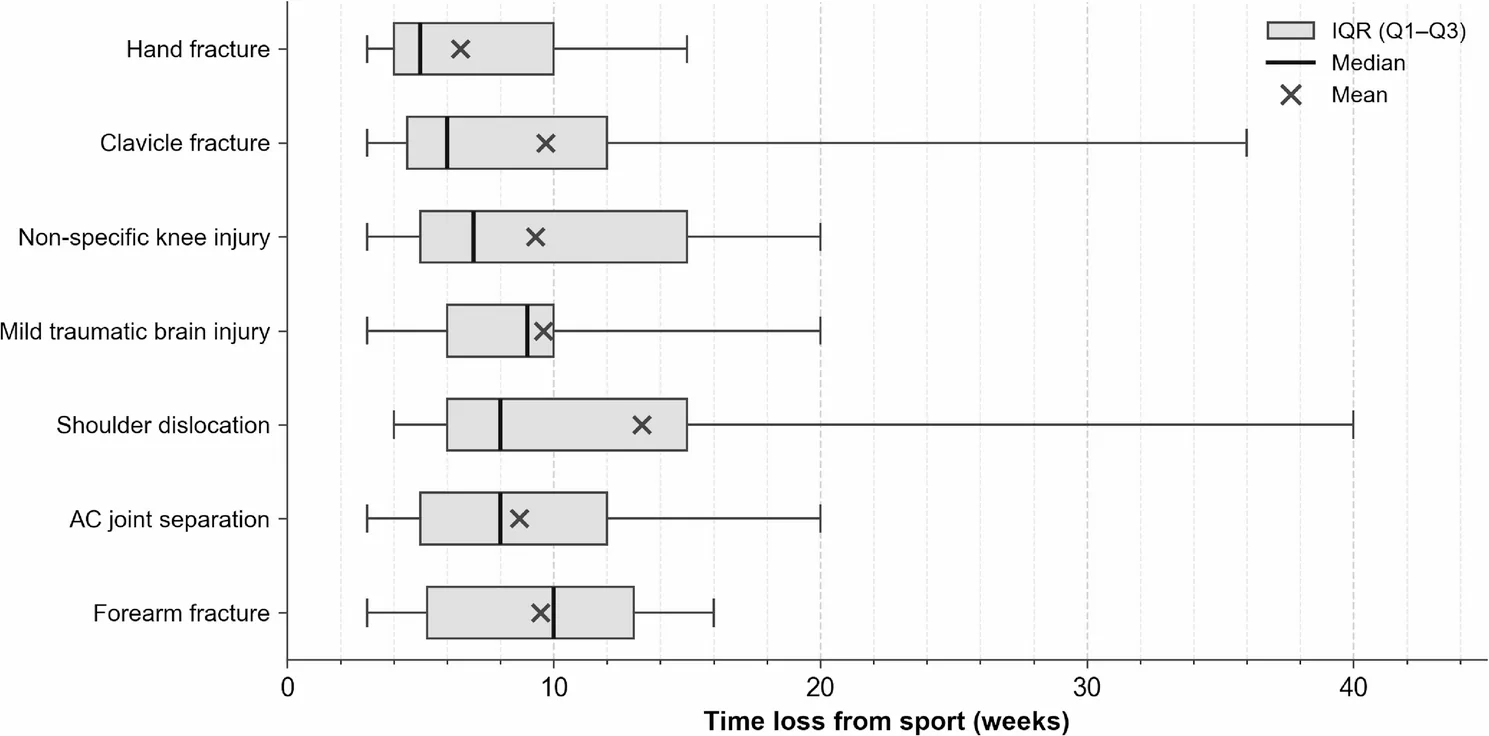
Time loss from sport after injury for the most common diagnoses. Boxes represent the interquartile range (IQR, from the 25th [Q1] to the 75th [Q3] percentile), the vertical line inside each box indicates the median, and the ×-symbol denotes the mean. Whiskers extend to the minimum and maximum values

The longest mean time loss was observed following shoulder dislocations (Fig. 5), with athletes being unable to compete for an average of 13.3 ± 12.5 weeks (95% CI 7.6–27.6; median: 8 weeks; range: 4–40 weeks). Mean time loss was also substantial following clavicle fractures (9.7 ± 9.2 weeks; 95% CI 6.4–17.3; median: 6 weeks; range: 3–36 weeks), mild traumatic brain injuries (9.6 ± 5.3 weeks; 95% CI 6.7–14.4; median: 9 weeks; range: 3–20 weeks), forearm fractures (9.5 ± 4.5 weeks; n = 6 with available data; 95% CI 6.2–12.7; median: 10 weeks; range: 3–16 weeks), and non-specific knee injuries (9.3 ± 6.1 weeks; 95% CI 6.2–13.9; median: 7 weeks; range: 3–20 weeks).

Shorter time loss was observed following AC joint separations (8.7 ± 5.8 weeks; 95% CI 5.7–14.3; median: 8 weeks; range: 3–20 weeks) and hand fractures (6.5 ± 3.5 weeks; 95% CI 5.1–8.8; median: 5 weeks; range: 3–15 weeks). Considerable variability in time loss was evident across injury types, particularly for shoulder dislocations and clavicle fractures, as reflected by the relatively large standard deviations.

### Return to pre-injury performance level

**Fig. 6:**
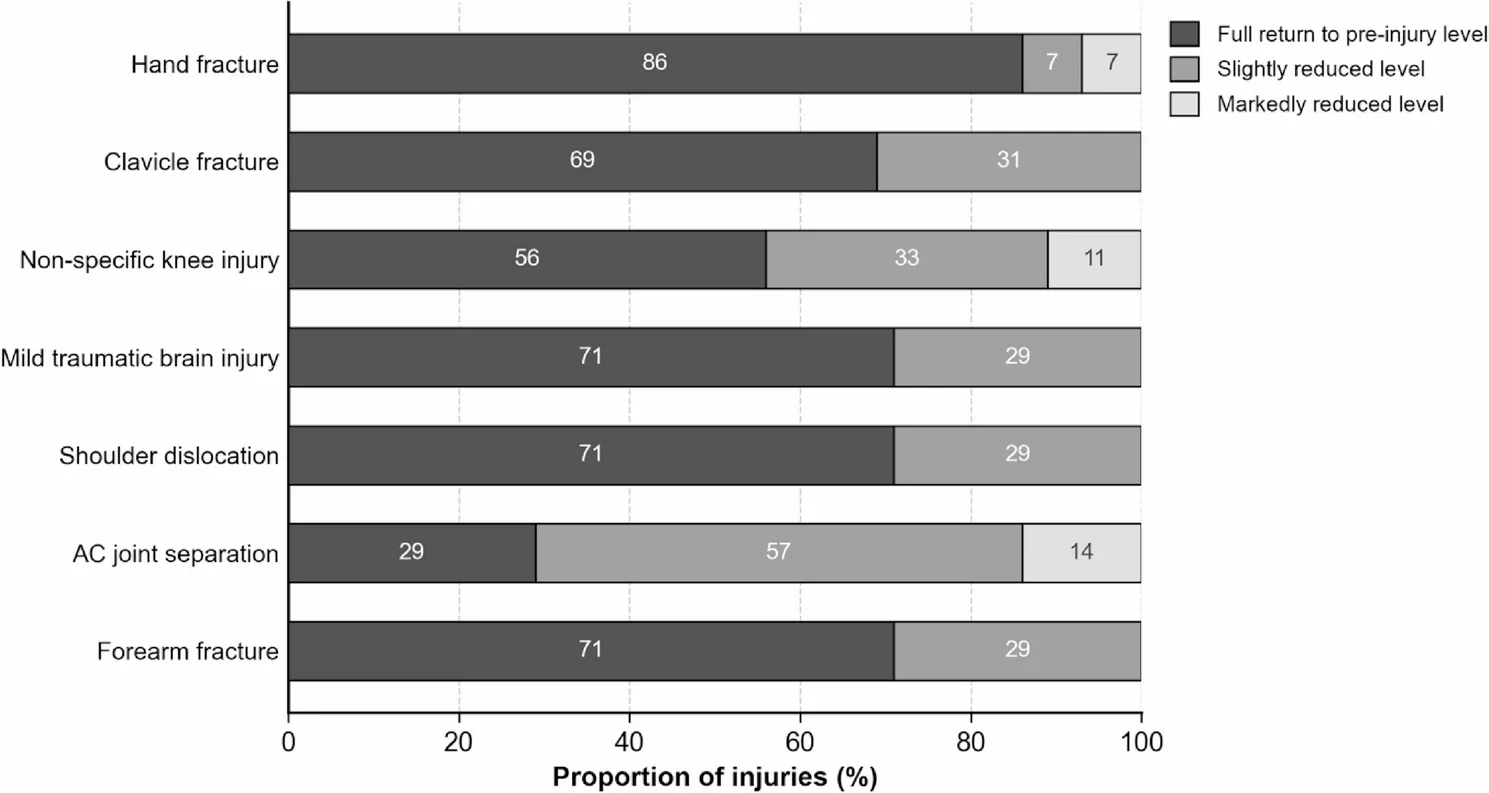
Performance level after return to sport for the seven most common diagnoses. Values are percentages of all injuries with the respective diagnosis; performance was self-reported as full return to the pre-injury level, slightly reduced level, or markedly reduced level

The highest rate of full return to the pre-injury performance level was observed after hand fractures (85.7%, n = 12; Fig. 6), while a slightly and a markedly reduced level were each reported in 7.1% (n = 1). Forearm fractures, mild traumatic brain injuries, and shoulder dislocations showed similar rates of full return (71.4%, n = 5 each). After clavicle fractures, 69.2% (n = 9) returned fully to their pre-injury level, while 30.8% (n = 4) reported a slightly reduced level. After non-specific knee injuries, a reduced performance level was reported in 44.4% (n = 4) of cases (33.3% slightly reduced, 11.1% markedly reduced). The lowest rate of full return was found after AC joint separations, with only 28.6% (n = 2) returning to their previous level; 57.1% (n = 4) reported a slightly reduced level and 14.3% (n = 1) a markedly reduced level.

## Discussion

This study provides an overview of severe injuries across all Olympic cycling disciplines in competitive and recreational athletes over one Olympic cycle. Of the 192 cyclists included, 72 (37.5%) reported at least one severe injury resulting in a time loss of at least three weeks, and a total of 237 injuries were analyzed. Upper extremity injuries were most common, with hand and clavicle fractures being the most frequent diagnoses. In exploratory analyses, hand fractures and shoulder dislocations were most frequent among BMX athletes, and hand fractures were more frequent in competitive than in recreational athletes. Clavicle fractures occurred predominantly during competition. Shoulder dislocations were associated with the longest time loss, whereas hand fractures showed the shortest time loss and the highest rate of full return to the pre-injury performance level. In contrast, AC joint separations were associated with the second shortest time loss but the lowest rate of full return, suggesting that time to return to sport does not necessarily reflect a full return to pre-injury performance.

### Injury location and patterns

The upper extremity was the most frequently injured body region (33.8%), followed by the lower extremity (27.8%), consistent with previous studies reporting a predominance of upper extremity injuries in cycling [8, 13]. Population-based data also demonstrate that upper extremity injuries occur more frequently than lower extremity injuries among cyclists, suggesting that this pattern is not restricted to competitive athletes [13]. Similarly, BMX-specific data identified the upper extremity as one of the most commonly affected regions despite substantial differences in discipline-specific demands and injury mechanisms [9]. A plausible explanation is the instinctive use of the arms and shoulders to break a fall, resulting in high impact forces on these structures [14].

Hand fractures and clavicle fractures were the most common injury diagnoses in the present cohort. This finding agrees with national registry data showing that fractures of the hand, forearm, and clavicle represent the most frequent fracture locations following cycling accidents [15]. Together, these studies suggest that traumatic upper extremity injuries represent a characteristic injury pattern across different cycling disciplines.

Shoulder injuries were common in both the present study and previous reports [8, 9]. In contrast, the proportion of head injuries was considerably lower than that reported in youth mountain bikers (7.6% vs. 23.3%)^8^. This discrepancy may reflect differences in study populations, cycling disciplines, terrain, and technical demands. The high proportion of traumatic upper extremity injuries observed in the present study underlines the importance of injury prevention strategies addressing fall mechanisms, protective equipment, and safe riding skills.

### Factors associated with injury patterns

No significant sex-related differences were observed for the most common injury diagnoses, consistent with the similar overall injury prevalence in female and male athletes. Although forearm fractures occurred exclusively in male athletes, this finding was based on small numbers and contrasts with bicycle-accident registry data reporting a higher proportion of forearm fractures among women than men [15]. Likewise, sex-specific injury patterns described in youth mountain bikers may not be directly transferable to our cohort of competitive and recreational cyclists [8].

In contrast, two diagnoses differed between cycling disciplines when expressed as the proportion of athletes within each discipline. Hand fractures were most frequent among BMX athletes (33.3%, compared with 8.1% of road cyclists), and shoulder dislocations were most frequent in BMX (20.0%) and did not occur in road cycling. These findings are consistent with previous studies identifying upper extremity injuries and fractures as characteristic injuries across cycling disciplines, particularly in BMX and mountain biking [4, 8, 9, 15]. The differences may reflect discipline-specific crash mechanisms, such as jumps, steep turns, and close contact between riders in BMX, although information on fall direction and collision characteristics was not available. Because the BMX group comprised only 15 athletes, these estimates are imprecise and should be regarded as exploratory.

Hand fractures were also more frequent in competitive athletes than in recreational athletes, possibly reflecting higher riding speeds and more demanding race and training situations. However, the proportion of competitive athletes differed between disciplines, ranging from 35.7% in mountain biking to 85.7% in track cycling. Differences between competitive levels may therefore partly reflect differences between disciplines, and vice versa. Given the small number of hand fractures (*n* = 14), the effects of discipline and competitive level could not be separated in this sample, and this finding should be interpreted with caution.

Finally, clavicle fractures occurred predominantly during competition, whereas non-specific knee injuries were mainly reported in training. Previous studies have described acute traumatic injuries as occurring mainly during competition and overuse injuries as being associated with training exposure [7, 14]. Because the mode of onset was not assessed in the present study, however, the training-related knee injuries cannot be classified as overuse injuries. These observations nevertheless support prevention strategies that take both the discipline and the injury setting into account.

### Time loss and return to performance

Shoulder dislocations resulted in the longest mean time loss (13.3 weeks), which may reflect the wide spectrum of injury severity and treatment strategies reported in the literature [16]. In contrast, hand fractures showed the shortest time loss (6.5 weeks) and the highest rate of full return to pre-injury performance (85.7%), consistent with previous studies demonstrating favorable outcomes following appropriate management of hand fractures in athletes [17, 18].

Clavicle fractures, one of the hallmark injuries in cycling, resulted in an intermediate time loss (9.7 weeks). Reported recovery times vary considerably depending on treatment. Operative management was associated with earlier return to competition in professional cyclists [19], and early return to athletic activity has also been described after plate fixation in a case series of professional and recreational cyclists [20]. The heterogeneous recovery observed in the present cohort may reflect differences in injury severity, treatment, and competitive level.

The lowest rate of full return to the pre-injury performance level was observed after AC joint separations, with only 28.6% of cases returning to their previous level despite a moderate mean time loss of 8.7 weeks. Although based on few cases, this contrasts with previous reports describing substantially higher return-to-sport rates after AC joint injuries [21]. The 2016 Bern consensus describes return to sport as a continuum from return to participation through return to sport to return to performance, the last stage being defined as performing at or above the pre-injury level [22]. The outcome assessed in the present study approximately corresponds to this final stage. The combination of moderate time loss and a low rate of return to performance after AC joint separations suggests that time to return alone does not establish whether athletes have regained their pre-injury performance level. Return to performance, rather than time to return alone, may therefore be a more meaningful target for rehabilitation after AC joint injuries in cyclists. Prospective studies are needed to confirm this finding.

## Limitations

This study has several limitations. Recruitment was open and relied on the project website, social media, and dissemination through national cycling federations, so the number of athletes reached could not be determined and a response rate could not be calculated. The reported figure of 37.5% therefore represents the proportion of respondents rather than a population prevalence, and self-selection bias cannot be excluded. The retrospective design over a period of up to five years is subject to recall bias, and self-reported diagnoses and outcomes were not clinically verified. No individual exposure data were available, which prevented the calculation of exposure-based injury rates and limited the comparison between competitive and recreational athletes. Because the questionnaire allowed only one entry per body region and year, the total number of injuries may be slightly underestimated. Finally, the number of events per diagnosis was small and several comparisons were performed, so the subgroup analyses are exploratory and are reported with 95% confidence intervals. Because of the small number of cases per diagnosis, return to performance could not be analyzed separately by discipline.

## Clinical implications

More than one-third of respondents reported at least one severe injury over the five-year period. Clavicle fractures occurred predominantly during competition, whereas non-specific knee injuries were mainly associated with training. These findings support the need for prevention strategies, including both crash prevention in competition and appropriate management of training load to prevent injuries.

Hand fractures and shoulder dislocations were most frequent among BMX athletes, suggesting that prevention programs may need to consider the specific demands of each discipline. While hand fractures were associated with a high rate of full return, AC joint separations were followed by a markedly lower rate of full return to the pre-injury performance level despite moderate time loss. This underlines the importance of distinguishing return to sport from return to performance when evaluating rehabilitation outcomes.

Future prospective studies should combine standardized injury surveillance with exposure-based data to quantify injury risk and to develop discipline-specific prevention strategies.

## Supplementary Information

Supplementary Material 1.

## Data Availability

Data sharing statementThe datasets generated and/or analyzed during the current study are not publicly available due to not finished analysis on other parts of this data, which are not published until this moment. But are available from the corresponding author on reasonable request.

## Abbreviation

AC: Acromioclavicular
BMX: Bicycle motocross
CI: Confidence interval
IQR: Interquartile range
SD: Standard deviation
