# Supplementary material for: Severe injuries in competitive cycling during an olympic cycle: injury patterns, time loss, and return-to-performance outcomes
Source: BMC Sports Sci Med Rehabil. 2026 Nov 3;18:558. doi: 10.1186/s13102-026-02201-x (PMC13737534; doi:10.1186/s13102-026-02201-x)
Supplement: Supplementary file 1 [file 13102_2026_2201_MOESM1_ESM.docx]

**Supplementary Appendix. Online questionnaire**

*The questionnaire was administered online in German (SurveyMonkey) as part of a multi-sport survey covering all Olympic sports. This appendix shows the items presented to cyclists, translated into English. Terms in square brackets were inserted automatically by the survey software ([sport] = the main sport selected by the participant; [year] = the respective calendar year). Unless stated otherwise, every item also offered the response option “no answer”.*

**Section A. Sport and screening**

**1. Which sport do you practise? (main sport only)**

*Selection from a list of all Olympic sports. Cycling disciplines were listed as track cycling, BMX, mountain biking, and road racing.*

**2. Have you been injured in [sport] during the past five years and had to take a competition break of more than three weeks as a result?**

☐ Yes

☐ No

*Questions 3 to 5 were asked separately for each year from 2012 to 2016.*

**3. What was your performance level in [year]? (multiple answers possible)**

☐ A squad

☐ B squad

☐ C squad

☐ D/C squad

☐ National league (Bundesliga)

☐ Recreational sport

☐ Sports student

☐ Professional coach

☐ No longer an athlete

**4. On average, how many hours per week did you train [sport] in [year]?**

______ hours per week

**5. Were you injured in [year]?**

☐ Yes

☐ No

**Section B. Injury history (repeated for each year from 2012 to 2016)**

*For each body region, participants first indicated whether they had injured this region in the respective year and whether the injury resulted in a competition break of more than three weeks. Only in that case were the region-specific follow-up questions shown.*

***B1. Head***

**6. Did you injure your head while [sport] in [year]?**

☐ Yes

☐ No

**7. Did you have to take a competition break of more than three weeks as a result?**

*Follow-up questions were shown only if this question was answered “yes”.*

☐ Yes

☐ No

**8. What type of head injury did you sustain?**

☐ Concussion

☐ Midface fracture (e.g., jaw, nose)

☐ Eye injury

**9. Did you sustain this injury during training or competition?**

☐ Training

☐ Competition

**10. Were you operated on because of this injury?**

☐ Yes

☐ No

**11. For how many weeks were you unable to compete because of this injury?**

Time loss: ______ weeks

**12. Were you able to regain your performance level after this injury?**

☐ Yes, fully

☐ Slightly reduced

☐ Markedly reduced

☐ No, I had to give up my sport

***B2. Shoulder***

**13. Did you injure your shoulder while [sport] in [year]?**

☐ Yes

☐ No

**14. Did you have to take a competition break of more than three weeks as a result?**

*Follow-up questions were shown only if this question was answered “yes”.*

☐ Yes

☐ No

**15. Which diagnosis was made? (multiple answers possible)**

☐ Not recalled

☐ Shoulder dislocation or subluxation

☐ Acromioclavicular joint separation (Rockwood/Tossy)

☐ Rotator cuff tear

☐ Clavicle fracture

☐ SLAP lesion

☐ Thoracic outlet syndrome

☐ Non-specific shoulder injury

**16. If acromioclavicular joint separation: which grade?**

☐ Tossy/Rockwood 1

☐ Tossy/Rockwood 2

☐ Tossy/Rockwood 3

☐ Rockwood 4

☐ Rockwood 5

☐ Rockwood 6

**17. Did you sustain this injury during training or competition?**

☐ Training

☐ Competition

**18. Were you operated on because of this injury?**

☐ Yes

☐ No

**19. If operated: how was the operation performed?**

☐ Arthroscopically

☐ Open

**20. For how many weeks were you unable to compete because of this injury?**

Time loss: ______ weeks

**21. Did you still have complaints after the injury break? (multiple answers possible)**

☐ No complaints

☐ Pain

☐ Instability

☐ Coordination problems

☐ Loss of strength

☐ Restricted range of motion

**22. Were you able to regain your performance level after this injury?**

☐ Yes, fully

☐ Slightly reduced

☐ Markedly reduced

☐ No, I had to give up my sport

**23. In [year], did you sustain another injury to the same shoulder after this injury?**

☐ Yes

☐ No

**24. In [year], did you sustain the same injury on the other side of the body, also resulting in a break of at least three weeks?**

*If “yes”, the follow-up questions were repeated for the contralateral side.*

☐ Yes

☐ No

***B3. Arm***

**25. Did you injure your arm while [sport] in [year]?**

☐ Yes

☐ No

**26. Did you have to take a competition break of more than three weeks as a result?**

*Follow-up questions were shown only if this question was answered “yes”.*

☐ Yes

☐ No

**27. Where on the arm were you injured?**

☐ Upper arm

☐ Elbow

☐ Forearm

☐ Hand

☐ Finger

**28. What type of injury did you sustain?**

☐ Fracture

☐ Muscle injury (strain, muscle fibre tear)

☐ Ligament injury

☐ Capsule injury

☐ Dislocation

**29. Did you sustain this injury during training or competition?**

☐ Training

☐ Competition

**30. Were you operated on because of this injury?**

☐ Yes

☐ No

**31. For how many weeks were you unable to compete because of this injury?**

Time loss: ______ weeks

**32. Were you able to regain your performance level after this injury?**

☐ Yes, fully

☐ Slightly reduced

☐ Markedly reduced

☐ No, I had to give up my sport

**33. In [year], did you sustain the same injury on the other side of the body, also resulting in a break of at least three weeks?**

*If “yes”, the follow-up questions were repeated for the contralateral side.*

☐ Yes

☐ No

***B4. Torso***

**34. Did you injure your torso while [sport] in [year]?**

☐ Yes

☐ No

**35. Did you have to take a competition break of more than three weeks as a result?**

*Follow-up questions were shown only if this question was answered “yes”.*

☐ Yes

☐ No

**36. Where on the torso were you injured?**

☐ Chest

☐ Ribs

☐ Abdomen

**37. What type of injury did you sustain?**

☐ Fracture

☐ Muscle injury (strain, muscle fibre tear)

☐ Ligament injury

☐ Capsule injury

☐ Cartilage injury

**38. Did you sustain this injury during training or competition?**

☐ Training

☐ Competition

**39. Were you operated on because of this injury?**

☐ Yes

☐ No

**40. For how many weeks were you unable to compete because of this injury?**

Time loss: ______ weeks

**41. Were you able to regain your performance level after this injury?**

☐ Yes, fully

☐ Slightly reduced

☐ Markedly reduced

☐ No, I had to give up my sport

***B5. Back***

**42. Did you injure your back while [sport] in [year]?**

☐ Yes

☐ No

**43. Did you have to take a competition break of more than three weeks as a result?**

*Follow-up questions were shown only if this question was answered “yes”.*

☐ Yes

☐ No

**44. Where on the back were you injured?**

☐ Cervical spine

☐ Thoracic spine

☐ Lumbar spine

☐ Sacrum

☐ Coccyx

**45. What type of injury did you sustain?**

☐ Fracture

☐ Muscle injury (strain, muscle fibre tear)

☐ Ligament injury

☐ Disc herniation

☐ Chronic back pain

**46. Did you sustain this injury during training or competition?**

☐ Training

☐ Competition

**47. Were you operated on because of this injury?**

☐ Yes

☐ No

**48. For how many weeks were you unable to compete because of this injury?**

Time loss: ______ weeks

**49. Were you able to regain your performance level after this injury?**

☐ Yes, fully

☐ Slightly reduced

☐ Markedly reduced

☐ No, I had to give up my sport

***B6. Leg or foot (except knee)***

**50. Did you injure your leg or foot (except the knee) while [sport] in [year]?**

☐ Yes

☐ No

**51. Did you have to take a competition break of more than three weeks as a result?**

*Follow-up questions were shown only if this question was answered “yes”.*

☐ Yes

☐ No

**52. Where on the leg were you injured?**

☐ Hip

☐ Thigh

☐ Fibula (lower leg)

☐ Tibia (lower leg)

☐ Foot

☐ Toes

☐ Ankle

**53. What type of injury did you sustain?**

☐ Fracture

☐ Muscle injury (strain, muscle fibre tear)

☐ Ligament injury

☐ Capsule injury

☐ Cartilage injury

**54. Did you sustain this injury during training or competition?**

☐ Training

☐ Competition

**55. Were you operated on because of this injury?**

☐ Yes

☐ No

**56. For how many weeks were you unable to compete because of this injury?**

Time loss: ______ weeks

**57. Were you able to regain your performance level after this injury?**

☐ Yes, fully

☐ Slightly reduced

☐ Markedly reduced

☐ No, I had to give up my sport

**58. In [year], did you sustain the same injury on the other side of the body, also resulting in a break of at least three weeks?**

*If “yes”, the follow-up questions were repeated for the contralateral side.*

☐ Yes

☐ No

***B7. Knee (except cruciate ligament)***

**59. Did you injure your knee (except the cruciate ligament) while [sport] in [year]?**

☐ Yes

☐ No

**60. Did you have to take a competition break of more than three weeks as a result?**

*Follow-up questions were shown only if this question was answered “yes”.*

☐ Yes

☐ No

**61. Did you sustain one or more of the following knee injuries? (multiple answers possible)**

☐ Not recalled

☐ Meniscus injury

☐ Medial collateral ligament injury

☐ Lateral collateral ligament injury

☐ Cartilage injury

☐ Non-specific knee injury (unclear diagnosis)

**62. Did you sustain this injury during training or competition?**

☐ Training

☐ Competition

**63. Were you operated on because of this injury?**

☐ Yes

☐ No

**64. If the meniscus was operated on: was it sutured, partially removed, or both?**

☐ Sutured

☐ Partially removed

☐ Both

**65. For how many weeks were you unable to compete because of this injury?**

Time loss: ______ weeks

**66. Did you still have complaints after the injury break? (multiple answers possible)**

☐ No complaints

☐ Pain

☐ Instability

☐ Coordination problems

☐ Loss of strength

☐ Restricted range of motion

**67. Were you able to regain your performance level after this injury?**

☐ Yes, fully

☐ Slightly reduced

☐ Markedly reduced

☐ No, I had to give up my sport

**68. In [year], did you sustain another injury to the same knee after this injury?**

☐ Yes

☐ No

**69. In [year], did you sustain the same injury on the other side of the body, also resulting in a break of at least three weeks?**

*If “yes”, the follow-up questions were repeated for the contralateral side.*

☐ Yes

☐ No

***B8. Cruciate ligament***

**70. Did you sustain a cruciate ligament rupture while [sport] in [year]?**

☐ Yes

☐ No

**71. Did you have to take a competition break of more than three weeks as a result?**

*Follow-up questions were shown only if this question was answered “yes”.*

☐ Yes

☐ No

**72. Which cruciate ligament was affected?**

☐ Anterior cruciate ligament

☐ Posterior cruciate ligament

**73. Did you sustain this injury during training or competition?**

☐ Training

☐ Competition

**74. Were you operated on because of this injury?**

☐ Yes

☐ No

**75. If operated: how much time passed between the injury and the operation?**

☐ Less than 48 hours

☐ 2 to 4 days

☐ 5 to 14 days

☐ 2 to 4 weeks

☐ 1 to 3 months

☐ 3 to 6 months

☐ 6 to 12 months

☐ More than 12 months

**76. If operated: which graft was used?**

☐ Semitendinosus/gracilis tendon

☐ Hamstrings (medial side of the knee)

☐ Patellar tendon (below the kneecap)

☐ Quadriceps tendon (above the kneecap)

☐ Other

**77. For how many weeks were you unable to compete because of this injury?**

Time loss: ______ weeks

**78. Did you still have complaints after the injury break? (multiple answers possible)**

☐ No complaints

☐ Pain

☐ Instability

☐ Coordination problems

☐ Loss of strength

☐ Pain at the graft harvest site

☐ Restricted range of motion

☐ Giving way

**79. Were you able to regain your performance level after this injury?**

☐ Yes, fully

☐ Slightly reduced

☐ Markedly reduced

☐ No, I had to give up my sport

**80. In [year], did you re-rupture the operated cruciate ligament?**

☐ Yes

☐ No

**81. In [year], did you also sustain a cruciate ligament rupture of the other knee, resulting in a break of at least three weeks?**

☐ Yes

☐ No

**Section C. Personal information**

**82. Are you male or female?**

☐ Male

☐ Female

**83. How old are you?**

______ years

**84. Have you ever participated in the Olympic or Paralympic Games?**

☐ Yes, in the year: ______

☐ No
